# Fire Extinguishers: A Point for Institutional Managers

**Published:** 1916-06-10

**Authors:** 


### FIRE EXTINGUISHERS: A POINT FOR INSTITUTIONAL MANAGERS.

A very important Report has been issued by the small but highly expert Home Office Committee appointed to investigate the merits of the various dry-powder fire extinguishers which are on the market. Three of these were selected for analysis at the National Physical Laboratory—namely, "Kyl-Fyre," "Galvo," and "Diamond." All three were found to contain a large proportion—about one-half—of sodium bicarbonate, with smaller quantities of calcium carbonate: their fire-smothering properties evidently depend on the evolution of carbonic-acid gas from these carbonates when subjected to high temperatures. "Kyl-Fyre" was selected for experimentation, although "Diamond" yields a slightly higher available percentage of carbonic acid. In view of the importance to institutional managers, especially in country districts, of taking every precaution possible for the immediate suppression of an outbreak of fire, the results of these experiments should be marked and digested by all to whom any degree of responsible management pertains. The immediate occasion of the Committee's appointment was the anticipated results of aerial bombardment; but the conclusions reached apply to any form of fire whatever, and the Home Office might well consider itself responsible for similar services to the community in peace as well as in war.

### The Tests and their Results.

By permission of the London County Council the tests were carried out at the London Fire Brigade Headquarters (the Chief Officer of the Brigade was one of the four members of the Committee) and in some condemned house property adjacent thereto. Small bombs were dropped from inconsiderable heights on to various heaps of inflammable materials in the open air and on to rooms containing rough imitations of inflammable furniture. The resulting conflagrations were allowed to proceed unhindered for short spaces of time, up to two or three minutes; and then various forms of extinguishers were called into play, amongst them being water in buckets, water in hose pipes, sand, liquid "extincteur" apparatus, and "Kyl-Fyre" dry-chemical extinguisher.

None of these was found to exercise "any material effect on the combustion of the bomb itself": but there was a marked general difference between the experiments in which water was used and those in which it was not. "The spread of the fire caused by the bomb was greatly limited, and in some cases totally prevented by the application of water; whereas after the application of the dry powder the fire continued to burn, although at each application some temporary check to the fire was noted."

Although water fails to extinguish the bomb itself in the earlier stages of combustion, it is far more effective than dry powder: this is attributed to the wetting of the surrounding material, which prevents the spread of the fire and confines it to the immediate neighbourhood of the bomb. Further, water is much the best means of putting out fires caused by the bombs. The theoretical basis of dry-powder extinguishers is also criticised, and it is pointed out that a vastly larger volume of steam is generated from a given quantity of water than of carbonic-acid gas from the same quantity of carbonates: steam, equally with $CO_2$, constitutes an atmosphere in which combustion does not take place.

### Buckets and Hose Compared.

The experiments showed that a given volume of water is more effective when sprayed from a hose than when thrown on from buckets by hand. This is a point in favour of "extincteur" apparatus; but the Committee add a caution as to the necessity of selecting reliable makes, as fatal accidents have occurred from the employment of those of faulty construction or unsuitable type. In this connection the Committee might well have been a little less reticent and have drawn up lists of those which they do and do not consider to be worthy of use; there seems to have been some lack of courage, more likely at the Home Office itself than amongst the members of the Committee.

The manipulation of the extinguishing agent was entrusted in the experiments to unskilled persons, a precaution which was obviously necessary in obtaining results applicable to the community at large.

In summing up their work the Committee very strongly deprecate the use of dry-powder extinguishers as being practically useless and as leading to a false sense of security. They further add a caustic note on the extortionate cost of dry-powder apparatus; the most expensive ingredient of the chemical powder they contain costs (wholesale) about a halfpenny a pound. Both from the point of view of cost and from that of efficiency in protection, the attention of those hospital boards, superintendents, and secretaries which have invested in dry-powder extinguishers should be very seriously directed to the Report of this Committee.

